# Epidemiological features and injury patterns of multiple fractures in children: a cross-sectional study

**DOI:** 10.3389/fpubh.2025.1709382

**Published:** 2025-10-29

**Authors:** Wei Gu, Haiyan Guo, Xu Wang, Yanqun Sun, Lei Ni

**Affiliations:** ^1^Clinical Medical Research Center, Children's Hospital of Nanjing Medical University, Nanjing, China; ^2^Department of Orthopedics, Children's Hospital of Nanjing Medical University, Nanjing, China

**Keywords:** multiple fracture, children, injury pattern, hierarchical clustering on principal components (HCPC), China

## Abstract

**Objective:**

This study aims to evaluate the epidemiological features of multiple pediatric fractures and identify specific injury patterns.

**Methods:**

This cross-sectional study retrospectively analyzed medical records for children with multiple fractures who presented to Children’s Hospital of Nanjing Medical University between 2016 and 2024. Fractures were categorized into six anatomical regions: Craniofacial, Trunk/Pelvis, Upper Limb, Lower Limb, Hand, and Foot. Patients were divided into single-region and multi-region groups based on the number of affected regions. Hierarchical clustering on principal components (HCPC) was applied to explore potential injury patterns.

**Results:**

Among the 1950 children with multiple fractures, 65.0% were boys, with a median age of 8.7 years (4.8–12.3 years). The primary mechanisms of injury included ground-level falls (36.3%), traffic accidents (13.8%), and play/sports activities (10.8%). Single-region fractures accounted for 77.2%, while multi-region fractures accounted for 22.8%. There were statistically significant differences in age and injury mechanism distributions between the two groups (*p* < 0.001). In the single-region group, children aged 6–11 years predominated, with ground-level falls being the most common cause (44.4%) primarily resulting in upper limb fractures (74.4%). The multi-region group had a higher representation of adolescents aged 12–17 years, with traffic accidents as the primary cause and injuries mainly involving the lower limbs (63.5%), trunk (56.8%), and upper limbs (50.5%). HCPC identified five distinct injury patterns: Lower Limb-Foot, Trunk/Pelvis-High Fall, Upper Limb-Hand, Craniofacial-Traffic Accident, and Upper Limb-Trunk/Pelvis patterns.

**Conclusion:**

Most pediatric multiple fractures involve a single anatomical region; however, the burden of cross-regional fractures should not be overlooked. Identifying potential injury patterns is crucial for developing targeted preventive strategies.

## Introduction

Pediatric traumatic fractures represent a significant global public health concern, profoundly impacting the physical and psychological well-being of children. Data from the Global Burden of Disease Study 2019 reveals an average age-standardized incidence rate (ASR) of 1,716.87 per 100,000 for fractures in children and adolescents worldwide (1). Epidemiological studies further underscore this issue, with pediatric fractures accounting for 32.6% of recorded injuries in children in China (2). More specifically, the National Nutrition and Health Systematic Survey for Children in China (2019–2021) (3) reported a 6.93% fracture incidence rate among children aged 6–17 years. While the peak incidence age varies slightly across studies due to regional and demographic differences, adolescents and school-aged children consistently exhibit the highest rates (1–4).

Fractures are generally considered common in the pediatric population, particularly among children engaging in vigorous physical activities. Children involved in high-intensity daily physical activities face a significantly elevated risk of fracture (5). Children’s strong bone remodeling capacity allows most cases to heal uneventfully (4, 6, 7). However, multiple fractures, due to their inherent complexity and high-energy injury mechanisms, can still impose substantial and long-lasting physical and psychological burdens on affected children (7). In adults, multiple fractures typically signify severe trauma, frequently co-occurring with other systemic injuries that demand immediate intervention and life support (8). Conversely, in children, a lack of self-preservation instincts coupled with active participation in vigorous sports and activities means that non-fatal multiple fractures are not uncommon (7). These injuries can lead to not only dysfunction and growth disturbances but also a spectrum of long-term complications (9). The repeated treatments and prolonged activity restrictions further exacerbate the psychological impact on children, intensifying the burden on their families.

Despite the prevalence of pediatric fractures, current epidemiological research lacks reliable incidence and characteristic data specifically concerning multiple fractures. Most studies do not mention multiple fractures or provide only brief notes on their occurrence (1, 2, 4). Existing clinical research primarily centers on specific bones and fracture types (e.g., distal radial fractures). Among the limited studies that do address multiple fractures, some merely discuss them as a subtopic within broader multiple trauma research (6, 10, 11), failing to delve into their unique epidemiological and clinical characteristics. Other studies primarily concentrate on multiple fractures associated with metabolic bone diseases or non-accidental injuries (12, 13).

This study aims to evaluate the epidemiological characteristics of pediatric multiple fractures at our center. By doing so, we intend to elucidate the features of multiple fractures across different age groups, gender distributions, and injury mechanisms. The findings from this research are expected to provide crucial evidence to inform the development of effective prevention strategies and targeted treatment protocols for pediatric multiple fractures.

## Methods

### Study design and data selection

This cross-sectional observational study received ethical approval from the Ethics Committee of Children’s Hospital of Nanjing Medical University (No. 202502020–1).

Medical records of patients diagnosed with fractures who presented to Children’s Hospital of Nanjing Medical University between June 1, 2016, and August 31, 2024, were retrospectively reviewed. Inclusion Criteria: (1) Children diagnosed with multiple traumatic fractures; (2) Initial presentation for the current fracture episode. Exclusion Criteria: (1) Pathological fractures; (2) Fractures associated with underlying bone development or metabolic disorders; (3) Fractures resulting from birth trauma; (4) Segmental fractures of a single bone; (5) Non-initial presentation; (6) Missing data for primary study variables.

Multiple fractures were defined as fractures occurring in two or more different bones upon admission. For statistical analysis, individual fractures were categorized into six anatomical regions: Craniofacial, Trunk/Pelvis, Upper Limb, Lower Limb, Hand, and Foot. Due to the small sample size of chest (*n* = 155), spine (*n* = 82), and pelvis (*n* = 108) fractures, these three categories were merged into Trunk/Pelvis. Hand and Foot fractures were kept separate from upper/lower limb categories given their distinct injury patterns and treatment strategies. All children were divided into two groups based on the number of affected anatomical regions: the single-region group and the multi-region group (involving two or more regions).

### Multiple correspondence analysis (MCA) and hierarchical clustering on principal components (HCPC)

Multiple correspondence analysis was utilized to assess the associations among the six fracture regions. MCA offers an exploratory method to evaluate relationships between categories of qualitative variables, visually representing them in a spatial map where proximity between variables indicates a potential association (14). The number of dimensions was determined based on the cumulative explained variance and the scree plot.

HCPC was employed to perform cluster analysis based on the MCA coordinates (15). The optimal number of clusters (k) was determined by examining dendrograms, within-cluster sum of squares (WSS), and silhouette coefficients. Each cluster was characterized by specific fracture patterns. Major cluster patterns were identified based on the distribution proportion of each fracture category within each cluster and their corresponding *V*-test values. Injury etiology and age groups were projected as supplementary variables onto the MCA map to aid interpretation. A *V*-test value greater than 1.96 indicated a significant expression of a variable category within a cluster compared to the overall distribution (*p* < 0.05). A higher *V*-test value signified greater representativeness of that category within the cluster (15).

### Enrichment analysis and co-occurrence analysis

For single-region fractures cases, enrichment analysis was conducted to evaluate the relative enrichment of specific injury Mechanisms within each anatomical region. The direction and strength of the effect were quantified using the binary logarithm transformation of the odds ratio (log2OR). Statistical significance was determined using Fisher’s exact test, with Benjamini–Hochberg (BH) correction applied for multiple comparisons.

For multi-region fracture cases, absolute co-occurrence frequencies and the Jaccard similarity coefficient were employed to comprehensively assess the co-occurrence patterns between anatomical regions. Statistical significance was also determined using Fisher’s exact test with BH correction.

### Statistical analysis

Continuous variables were presented as mean ± standard deviation or median (interquartile range), depending on the results of normality tests. Comparisons between groups were performed using Student’s *t*-test or the Mann–Whitney *U* test. Categorical variables were expressed as frequencies (percentages), and inter-group comparisons were conducted using the Chi-square test or Fisher’s exact test. A two-sided *p*-value < 0.05 was considered statistically significant. Adjusted *p*-values were reported for enrichment and co-occurrence analyses. All data analyses were performed using R software (version 4.5.0), with the FactoMineR package used for MCA and HCPC.

## Results

This study included a total of 1950 pediatric fracture patients, with 1,506 (77.2%) in the single-region group and 444 (22.8%) in the multi-region group. Males constituted 65.0% of the total study population, with no statistically significant difference in gender distribution between the two groups (*p* = 0.911). The median age of all patients was 8.7 (4.8–12.3) years, with the 6–11 age group representing the largest proportion (44.1%). The single-region group had a higher proportion of 6–11 year olds (45.9%), whereas the multi-region group showed a higher proportion of patients aged 12–17 years (28.2%). There was a statistically significant difference in age distribution between the two groups (*p* < 0.001) (Table 1).

**Table 1:** Demographic and clinical characteristics of children with multiple fractures.

Charac.	All (*N* = 1950)	Single-region (*n* = 1,506)	Multi-region (*n* = 444)	*P-*value
Age (Y)	8.7 (4.8–12.3)	8.2 (4.6–12.1)	11.4 (7.3–12.9)	**0.001**
Sex				0.911
Male	1,267 (65.0)	980 (65.1)	287 (64.6)	
Female	683 (35.0)	526 (34.9)	157 (35.4)	
Age group (Y)				**<0.001**
0–2	258 (13.2)	199 (13.2)	59 (13.3)	
3–5	503 (25.8)	410 (27.2)	93 (20.9)	
6–11	859 (44.1)	692 (45.9)	167 (37.6)	
12–17	330 (16.9)	205 (13.6)	125 (28.2)	
Season				0.447
Autumn	551 (28.3)	439 (29.2)	112 (25.2)	
Spring	541 (27.7)	411 (27.3)	130 (29.3)	
Summer	550 (28.2)	420 (27.9)	130 (29.3)	
Winter	308 (15.8)	236 (15.7)	72 (16.2)	
Mechanisms				**<0.001**
Collision/Crush	58 (3.0)	39 (2.6)	19 (4.3)	
Ground fall	708 (36.3)	669 (44.4)	39 (8.8)	
High fall	121 (6.2)	38 (2.5)	83 (18.7)	
Play/Sport	210 (10.8)	150 (10.0)	60 (13.5)	
Traffic accident	270 (13.8)	124 (8.2)	146 (32.9)	
Unspecified	583 (29.9)	486 (32.3)	97 (21.8)	
Site				–
Upper Limb	1,345 (69.0)	1,121 (74.4)	224 (50.5)	
Lower Limb	452 (23.2)	170 (11.3)	282 (63.5)	
Craniofacial	113 (5.8)	62 (4.1)	51 (11.5)	
Hand	63 (3.2)	27 (1.8)	36 (8.1)	
Foot	146 (7.5)	33 (2.2)	113 (25.5)	
Trunk/Pelvis	345 (17.7)	93 (6.2)	252 (56.8)	

Due to potential double counting within the multi-region cases, group comparisons for fracture regions were not performed.Statistically significant results (*p* < 0.05) are in bold.

The primary injury mechanisms included ground-level falls (36.3%), traffic accidents (13.8%), and play and sports-related incidents (10.8%). High-level collision and crush injuries were relatively less common. Unspecified causes accounted for 29.9% (*n* = 583). The distribution of injury mechanisms differed significantly between the two groups (*p* < 0.001). Ground-level falls primarily caused single-region fractures (44.4%), while multi-region fractures were relatively more often attributed to traffic accidents (32.9%) and high-level falls (18.7%). A sensitivity analysis, conducted after excluding individuals with “unspecified causes,” yielded results consistent with the primary analysis (Supplementary Table S1). Due to potential double counting in the multi-region group, inter-group significance testing was not performed for this variable.

In the single-region group, Upper Limb fractures were the most common (74.4%), followed by Lower Limb fractures (11.3%). In contrast, the multi-region group showed a primary involvement pattern of Lower Limb (63.5%), Trunk/Pelvis (56.8%), and Upper Limb (50.5%) fractures.

### Mechanisms and fracture regions in single-region fractures

**Figure 1:**
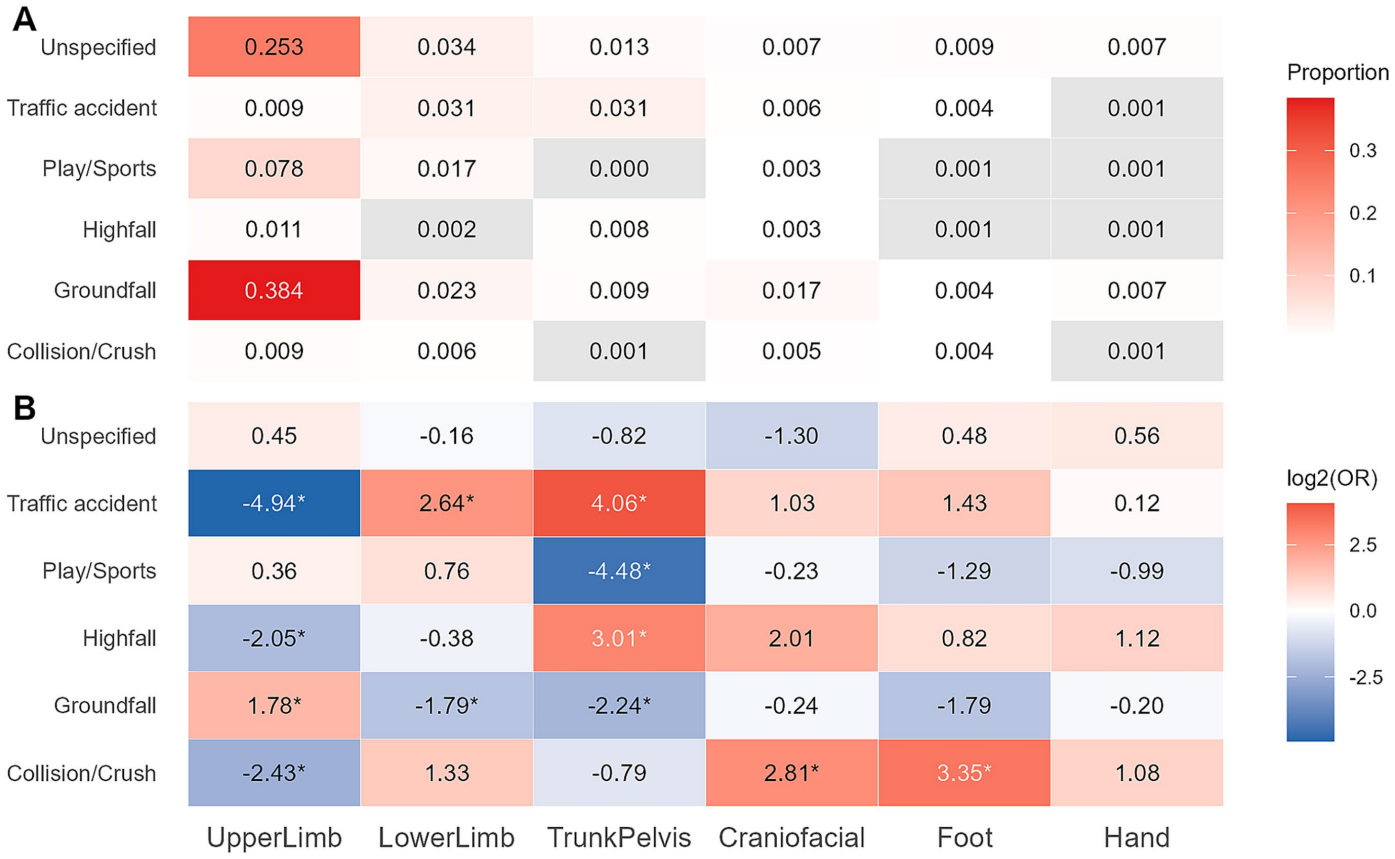
Association between injury mechanisms and fracture regions in children with single-region fractures. **(A)** Proportion of mechanism-region combinations within the entire study population. **(B)** Heatmap of mechanism-region enrichment. Cell values represent the degree of enrichment or depletion of a specific fracture region associated with each injury mechanism, relative to its overall prevalence in the single-region group. Fisher’s exact test with Benjamini–Hochberg (BH) adjustment for *p*-values was used. *p < 0.05.

We analyzed the association between fracture regions and injury mechanisms in the single-region group (Figure 1). Figure 1A illustrates the proportion of specific cause-region combinations within the entire study population, with gray cells indicating a co-occurrence frequency of less than 5. Figure 1B further demonstrates the relative distribution of injury mechanisms for each fracture region. For instance, Traffic accident-related fractures were significantly enriched in Lower Limb (log₂OR = 2.64, *p* < 0.05) and Trunk/Pelvis (log₂OR = 4.06, *p* < 0.05), but significantly negatively associated with Upper Limb (log₂OR = -4.94, *p* < 0.05).

### Co-occurrence analysis of multi-region fractures

**Figure 2:**
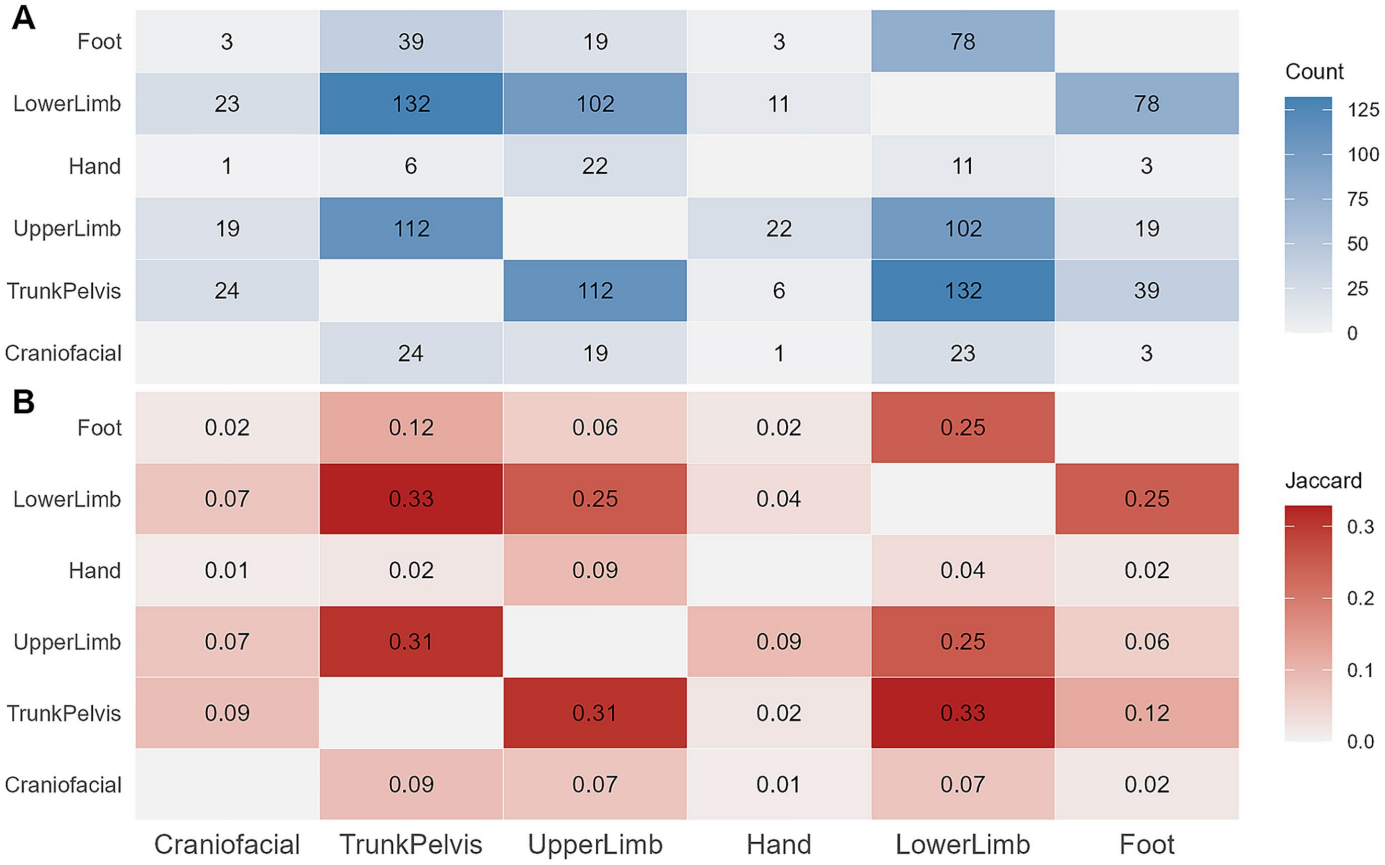
Co-occurrence of fracture regions in children with multi-region fractures. **(A)** Frequency of co-occurring combinations. **(B)** Jaccard similarity coefficients. Fisher’s exact test with Benjamini–Hochberg (BH) adjustment for *p*-values was used.

Among the 444 children with multi-region fractures, pairwise co-occurrence of the six anatomical regions was analyzed. Figure 2A illustrates the frequency of co-occurrence between regions. The most frequent co-occurrence patterns were Trunk/Pelvis with Lower Limb fractures (*n* = 132) and Trunk/Pelvis with Upper Limb fractures (*n* = 112).

Jaccard similarity coefficient analysis (Figure 2B), which mitigates potential interference from absolute frequencies, further quantified the standardized strength of co-occurrence. Trunk/Pelvis with Lower Limb (Jaccard = 0.328) fractures were identified as the combinations with the strongest co-occurrence, followed by Trunk/Pelvis with Upper Limb (Jaccard = 0.308) fractures.

**Figure 3:**
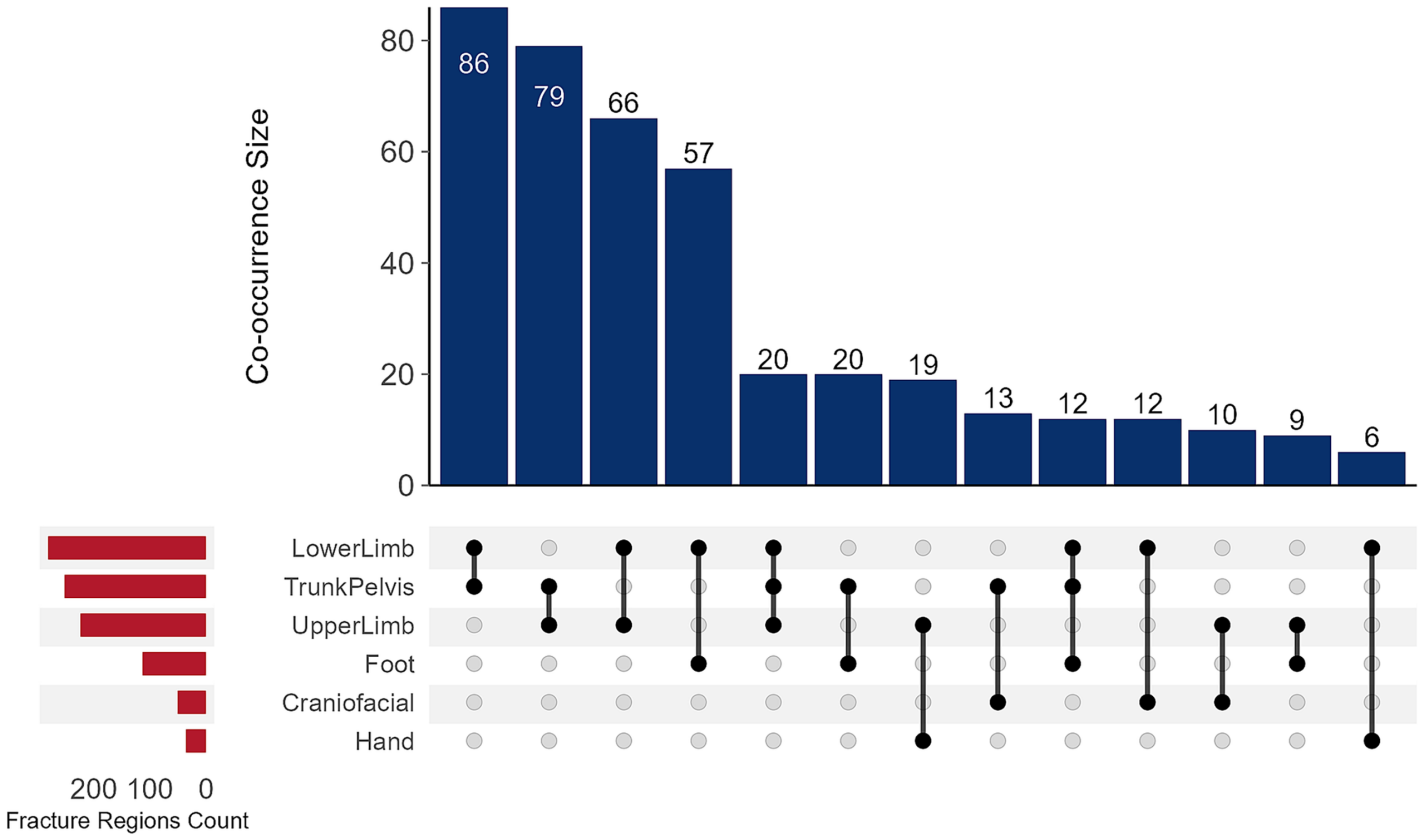
UpSet plot of co-occurrence patterns among different fracture regions.

To further characterize specific injury patterns in children with multi-region fractures, the frequencies of all fracture combinations were tabulated. Figure 3 presents all 13 combinations with frequency counts greater than 5. The analysis revealed that injury patterns involving two anatomical regions (*n* = 2) were the most common form of multi-region fractures. The most frequent combination was simultaneous Trunk/Pelvis and Lower Limb fractures (*n* = 86, 19.4%). Combinations involving three or more anatomical regions (*n* ≥ 3) were notably less common, with the most frequent pattern being involvement of Trunk/Pelvis, Upper Limb, and Lower Limb regions (*n* = 20, 4.5%). Only 4 patients (0.9%) sustained fractures in four regions, and only 1 patient (0.2%) presented fractures in five regions.

### MCA

**Figure 4:**
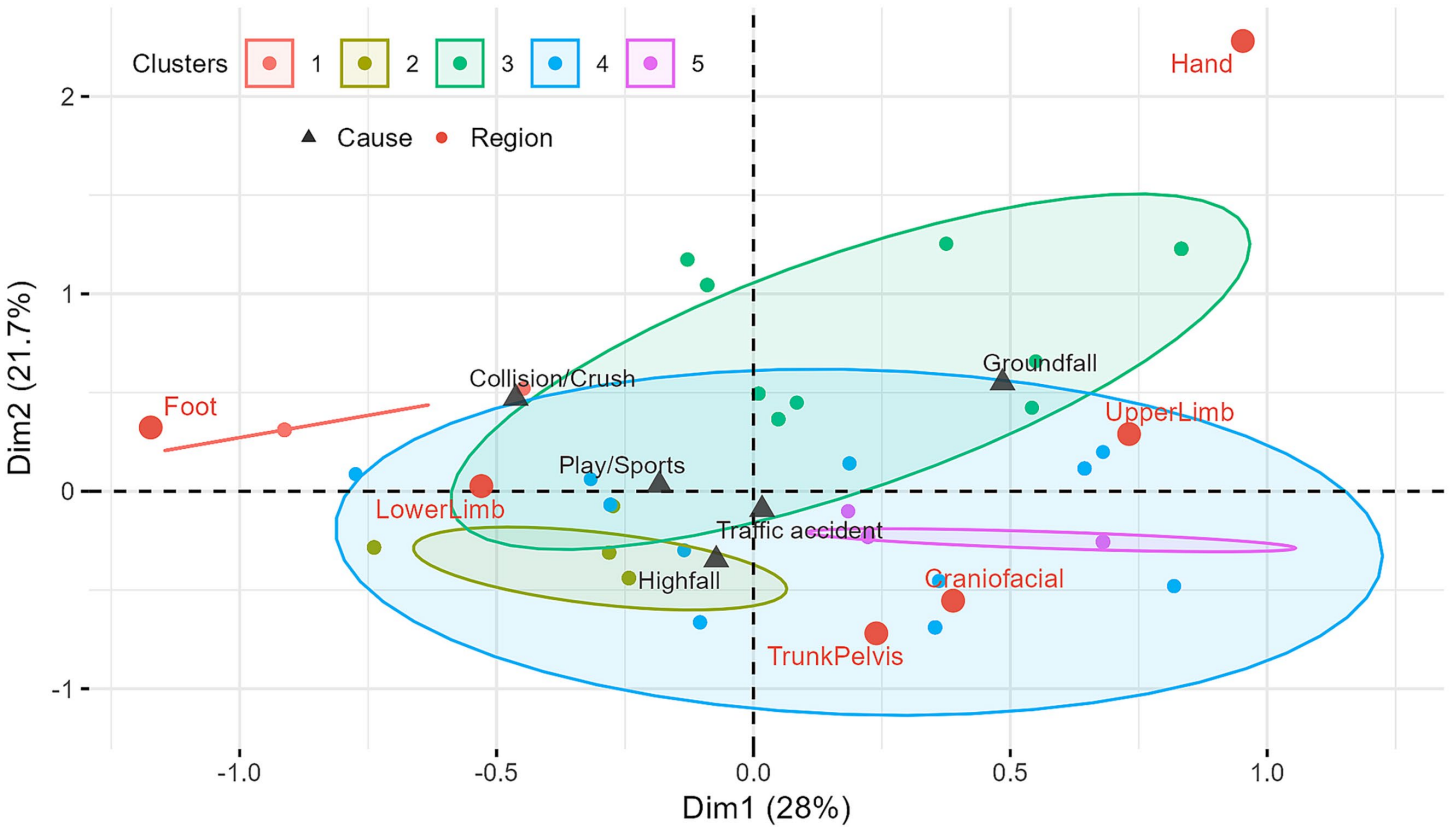
Biplot of multiple correspondence analysis (MCA). Clusters 1–5 represent groups of children with similar fracture patterns, and the ellipses indicate the 95% confidence regions for the distribution of these individuals. Proximity between variable points, as well as between variable points and clusters, implies an association.

The MCA explained the total variance of the data across 6 dimensions. The first dimension accounted for 28.0% of the total variance, the second for 21.7%, and the third for 17.6%, with the first three dimensions cumulatively explaining 67.3% of the total variance (Supplementary Figure S1). Figure 4 illustrates the distribution of different fracture regions and injury mechanisms (supplementary variables) on the first two dimensions of the MCA map.

Based on the association and contribution of each variable category to the respective dimensions, we observed that Dimension 1 was primarily defined by Upper Limb fractures, Lower Limb fractures, and Foot fractures. Dimension 2 was mainly defined by the Trunk/Pelvis and Hand fractures, while the Craniofacial fractures dominated the formation of Dimension 3 (Supplementary Table S2). Individuals with Upper Limb fractures tended to appear on the right side of the graph (positive direction of Dim1), while those with Lower Limb and Foot fractures tended to appear on the left side (negative direction of Dim1). Individuals with Trunk/Pelvis and Hand fractures tended to appear on the bottom (negative direction of Dim2) and top (positive direction of Dim2) of the graph, respectively. Craniofacial fractures had lower representation in the first two dimensions, tending to appear in the positive direction of Dim3 (Supplementary Figure S2).

Injury mechanisms were mapped as supplementary variables onto the MCA map (Figure 4). From the first two dimensions, ground-level fall showed a strong association with Upper Limb fractures (proximity to the Upper Limb point), play and sports-related incident were associated with Lower Limb fractures, and collision and crush injurie were associated with Lower Limb and Foot fractures. High-level falls were closer to Lower Limb and Trunk/Pelvis fractures. Traffic accident appeared near the origin on the first two dimensions, while on Dim3, it was associated with Trunk/Pelvis and Craniofacial fractures.

### HCPC

The 444 children with multi-region fractures were grouped into 5 clusters, with a relatively balanced distribution of cases and no extremely small clusters (*n* < 44, 10% of total). Figure 4 depicts the distribution of the 5 clusters on the first two dimensions of the MCA map.

Cluster 1 (*n* = 60): Lower Limb-Foot Pattern. All patients in this cluster had Foot fractures (100%, *v*-test = 13.26) and Lower Limb fractures (100%, *v*-test = 5.87). Upper Limb fractures were significantly absent (95%, *v*-test = 7.04). Trunk/Pelvis, Hand, and Craniofacial regions were not involved at all. Traffic accidents accounted for 30% of fractures, and collision and crush injuries were significantly enriched (11.7% vs. 4.2%, *v*-test = 2.83). Only 4 patients (6.7%) were aged 0–2 years, with other age groups relatively evenly distributed.

Cluster 2 (*n* = 122): Trunk/Pelvis-High Fall Pattern. All patients in this cluster had Trunk/Pelvis fractures (100%, *v*-test = 4.61), and 83.6% had Lower Limb fractures (*v*-test = 13.26). Upper Limb fractures were significantly absent (96.7%, *v*-test = 10.42), and Foot fractures were absent in 70.5%. Hand and Craniofacial regions were not involved at all. High-level falls were significantly enriched (30.3% vs. 18.7%, *v*-test = 3.30), and 33.3% of patients were aged 3–5 years.

Cluster 3 (*n* = 110): Upper Limb-Hand Pattern. There was a significant enrichment of Upper Limb (88.2%, *v*-test = 7.91) and Hand fractures (31.8%, *v*-test = 9.11). Trunk/Pelvis (95.5%, *v*-test = 11.05) and Foot (89.1%, *v*-test = 3.50) involvement were significantly absent, and Craniofacial region was not involved at all. Traffic accidents accounted for 32.7%, and play/sports activities were relatively prominent (17.3% vs. 13.5%). Males were significantly represented (73.6% vs. 64.6%, *v*-test = 1.97), and adolescents aged 12–17 years constituted a higher proportion (36.3% vs. 28.2%, *v*-test = 1.91). 34.5% of cases occurred in summer.

Cluster 4 (*n* = 51): Craniofacial-Traffic Accident Pattern. All patients in this cluster had Craniofacial fractures (100%, *v*-test = 19.82). Foot (94.1%, *v*-test = 3.21) and Lower Limb (54.9%, *v*-test = 2.73) involvement were significantly absent. Traffic accidents were significantly enriched (66.7%, *v*-test = 5.14), and patients with ≥3 involved regions accounted for 29.4% (*v*-test = 3.25).

Cluster 5 (*n* = 101): Upper Limb-Trunk/Pelvis Pattern. All patients in this cluster had Upper Limb (100%, *v*-test = 9.96) and trunk fractures (100%, *v*-test = 8.77). Foot (98.0%, *v*-test = 5.42) and Lower Limb (80.2%, *v*-test = 9.13) involvement were significantly absent, and Craniofacial and Hand regions were not involved at all. Traffic accidents and high-level falls accounted for 25.7 and 21.8%, respectively. Patients aged 0–2 years were relatively significant (16.8% vs. 7.2%, *v*-test = 3.74), and 21.8% of patients had ≥3 involved regions (*v*-test = 2.35).

## Discussion

This study leveraged a substantial cross-sectional dataset of 1950 patients to delineate the complicated patterns of pediatric multiple fractures. Our findings reveal that while most pediatric fractures concentrate within a single anatomical region (77.2%), the burden of cross-regional multiple fractures remains significant, impacting nearly a quarter of the study population (22.8%). Fractures were more prevalent in boys (65.0%) and school-aged children (44.1%), with a relatively higher proportion of adolescents (28.2%) observed in multi-region fracture cases. Through MCA and HCPC, we identified five distinct and clinically meaningful injury patterns. These findings provide data support for understanding the mechanisms and high-risk populations leading to multiple fractures.

The incidence of pediatric fractures is intimately linked to both sex and age. Consistent with our findings, numerous large-scale cross-sectional screening studies (2, 16, 17) have reported a male predominance among children with fractures. This global phenomenon, where male children experience higher fracture rates, likely stems from their increased participation in vigorous physical activities and differential skeletal development during adolescence (3, 16). The age distribution of fractures is influenced by various factors, including regional variations, socioeconomic status, and growth patterns. Consequently, variations in the peak age of fracture incidence have been observed across different studies (2, 3, 16, 17). Nevertheless, school-aged children (6–11 years) and adolescents (12–17 years) consistently emerge as the primary populations affected by fractures, aligning with the observations in our study.

Our results indicate that Upper Limb fractures overwhelmingly dominate among children with multiple fractures (69.0%). This finding resonates with global epidemiological trends (3, 17, 18). Fractures of the long bones of the Upper Limb have long constituted the majority of pediatric fractures, with the distal radius and ulna being the most common sites, and the distal humerus also frequently involved. Ground-level falls (36.3%), traffic accidents (13.8%), and play/sports-related incidents (10.8%) represent the predominant injury mechanisms, consistent with other literature (3, 17). We further explored the association between fracture regions and injury mechanisms. Our analysis revealed that ground-level falls are primarily associated with Upper Limb fractures. These low-energy injuries often lead to a fall onto an outstretched hand (FOOSH), a protective reflex that transmits impact forces to the forearm, rendering it highly vulnerable (19). In contrast to low-energy falls, high-energy mechanisms are less common in single-region fracture cases and manifest in distinct fracture patterns. High-level falls are significantly associated with Trunk/Pelvis fractures, suggesting severe axial compressive forces upon impact. Traffic accidents, another major cause of high-energy injuries, showed a strong association with Lower Limb and Trunk/Pelvis fractures. Our data underscore that, even within the context of multiple fractures, low-energy injuries resulting in fractures confined to a single anatomical region remain prevalent, demonstrating some consistency with the distribution patterns of isolated fractures.

A clear divergence in injury mechanisms between the single-region and multi-region groups is evident. In children with multi-region fractures, the dominant factors shift to traffic accidents (23.9%) and high-level falls (18.7%). Similarly, we observe differing trends in fracture regions distribution between the two groups, with Lower Limb and Trunk/Pelvis fractures diluting the predominance of Upper Limb fractures. Analysis of fracture co-occurrence in children with multi-region injuries provided insights into common injury pairings. The data suggests that the most frequent and strongly associated combination is Trunk/Pelvis with Lower Limb fractures (132 cases, Jaccard = 0.33). Simultaneous injury to the Trunk/Pelvis and Lower Limb is a classic presentation in pediatric traffic accidents (20), often termed “bumper injuries,” where the primary impact frequently affects the child’s pelvis and lower extremities.

Cross-anatomical region multiple fractures in children invariably imply high-energy injury mechanisms and highly correlated force transmission pathways. Clinical reality suggests that traumatic multiple fractures in children are multifaceted and complex injury combinations, where simple descriptive characterizations may fail to fully capture and elucidate their injury patterns. Therefore, this study employed HCPC to gain a more comprehensive understanding of the intrinsic patterns of multiple fractures. HCPC segmented the multi-region fracture population into five distinct clusters, each representing a unique fracture pattern linked to specific mechanisms and demographic characteristics. A clear overarching theme is the impact of high-energy trauma on Lower Limb and axial skeleton. Cluster 1 (Foot-Lower Limb Pattern) is almost entirely defined by Foot and Lower Limb fractures. Although strongly associated with crush/compression mechanisms, the overall number of cases due to this specific mechanism is quite limited. The 30% contribution, combined with the potential proportion within unspecified causes, leads us to hypothesize that this group might represent patients with Lower Limb involvement due to traffic accidents. Complementing this is Cluster 2 (Trunk/Pelvis-High Fall Pattern), characterized by Trunk/Pelvis fractures, often with concomitant Lower Limb involvement, and significantly enriched for high-level falls, reflecting severe vertical deceleration forces. Together, these two clusters identify a specific phenotype of high-energy trauma concentrated in the lower body.

In contrast, other clusters highlight upper-body-centric injury patterns. Cluster 3 (Upper Limb-Hand Pattern) captures a common clinical scenario: Upper Limb and Hand fractures resulting from traffic accidents and sports, particularly in older boys. This is especially pertinent in bicycle-related road accidents, where an instinctive self-protective fall leads to the forearm and hand bearing the brunt of the impact (21). Another upper-body dominant pattern is observed in Cluster 5 (Upper Limb-Trunk/Pelvis Pattern), which includes concurrent Upper Limb and Trunk/Pelvis fractures and is overrepresented in younger children, possibly reflecting different biomechanical responses to trauma in infants and toddlers. Finally, Cluster 4 (Craniofacial-Traffic Accident Pattern) delineates a severe injury phenotype characterized by Craniofacial fractures strongly associated with traffic accidents. The high frequency of involvement of three or more fracture regions in this cluster underscores the substantial energy transfer inherent in these events, often accompanied by secondary head impacts, potentially related to being thrown against vehicles or the ground (22). Although we were unable to separately analyze spinal fractures due to limited case numbers, it is a clinical imperative that in such high-energy events, a high index of suspicion for associated cervical spine injuries is maintained.

This study successfully identified five distinct groups of children with multiple fractures using HCPC. While our findings offer preliminary insights into potential patterns among children with multiple fractures, the study is not without limitations. This study was conducted at a single tertiary center in Nanjing, which may introduce selection bias; our patient population might not represent the broader pediatric population in China, limiting the generalizability of the findings. Due to sample size constraints, fractures of the chest, spine, and pelvis were consolidated, which may introduce bias into pattern recognition. Furthermore, unspecified mechanisms in some cases partially hampered the interpretation of clustered phenotypes. Nevertheless, these limitations do not fully negate the exploratory value of this research. In future studies, incorporating systematic fracture classifications (such as AO/OTA) together with detailed analyses of injury mechanisms (e.g., Injury Severity Score – ISS) could help delineate a more comprehensive model of pediatric multiple fractures, thereby enriching the epidemiological context for their diagnosis and treatment.

## Conclusion

This study demonstrates that while pediatric multiple fractures predominantly involve a single anatomical region, the burden of cross-regional fractures remains substantial. This research offers novel perspectives on understanding the epidemiological characteristics of pediatric multiple fractures and provides a basis for developing targeted prevention strategies. Encouraging the use of helmets and child safety restraints, along with implementing injury prevention programs in schools—such as training in self-protection during falls and promoting the use of protective equipment—can effectively help prevent multiple fractures in children.

## Data Availability

The raw data supporting the conclusions of this article will be made available by the authors, without undue reservation.
