# Supplementary material for: Epidemiological features and injury patterns of multiple fractures in children: a cross-sectional study
Source: Front Public Health. 2025 Oct 29;13:1709382. doi: 10.3389/fpubh.2025.1709382 (PMC12605433; doi:10.3389/fpubh.2025.1709382)
Supplement: Supplementary file 1 [file Data_Sheet_1.docx]

Table S1 Demographic and Clinical Characteristics of Children with Multiple Fractures.

| Charac. | All  (N=1367) | Single-region (n=1020) | Multi-region （n=347） | *P-*value |
| --- | --- | --- | --- | --- |
| Sex |  |  |  | 0.911 |
| Male | 900 (65.8) | 670 (65.7) | 230 (66.3) |  |
| Female | 467 (34.2) | 350 (34.3) | 117 (33.7) |  |
| AgeGroup (Y) |  |  |  | **<0.001** |
| 0-2 | 151 (11.0) | 118 (11.6) | 33 (9.5) |  |
| 3-5 | 350 (25.6) | 279 (27.4) | 71 (20.5) |  |
| 6-11 | 609 (44.6) | 473 (46.4) | 136 (39.2) |  |
| 12-17 | 257 (18.8) | 150 (14.7) | 107 (30.8) |  |
| Season |  |  |  | 0.447 |
| Autumn | 373 (27.3) | 294 (28.8) | 79 (22.8) |  |
| Spring | 389 (28.5) | 291 (28.5) | 98 (28.2) |  |
| Summer | 390 (28.5) | 281 (27.5) | 109 (31.4) |  |
| Winter | 215 (15.7) | 154 (15.1) | 61 (17.6) |  |
| Mechanisms |  |  |  | **<0.001** |
| Collision/Crush | 58 (3.0) | 39 (3.8) | 19 (5.5) |  |
| Ground fall | 708 (36.3) | 669 (65.6) | 39 (11.2) |  |
| High fall | 121 (6.2) | 38 (3.7) | 83 (23.9) |  |
| Play/Sports | 210 (10.8) | 150 (14.7) | 60 (17.3) |  |
| Traffic accident | 270 (13.8) | 124 (12.2) | 146 (42.1) |  |

Table S2 Association and Contribution of Fracture Regions to the First Three Dimensions of Multiple Correspondence Analysis (MCA).

|  | **Dim 1** | | **Dim 2** | | **Dim 3** | |
| --- | --- | --- | --- | --- | --- | --- |
|  | v-test | contribution | v-test | contribution | v-test | contribution |
| Craniofacial | 2.95 | 1.03 | -4.2 | 2.71 | 20.07 | 76.14 |
| Trunk/Pelvis | 5.77 | 1.93 | -17.35 | 22.59 | -6.83 | 4.31 |
| Upper Limb | 15.53 | 16.08 | 6.16 | 3.26 | -3.02 | 0.97 |
| Hand | 5.96 | 4.39 | 14.26 | 32.43 | -0.32 | 0.02 |
| Lower Limb | -14.71 | 10.62 | 0.73 | 0.03 | 0.91 | 0.06 |
| Foot | -14.43 | 20.89 | 3.97 | 2.04 | -2.95 | 1.38 |

A v-test value >1.96 indicated a significant expression of a variable category within a cluster compared to the overall distribution (P < 0.05).


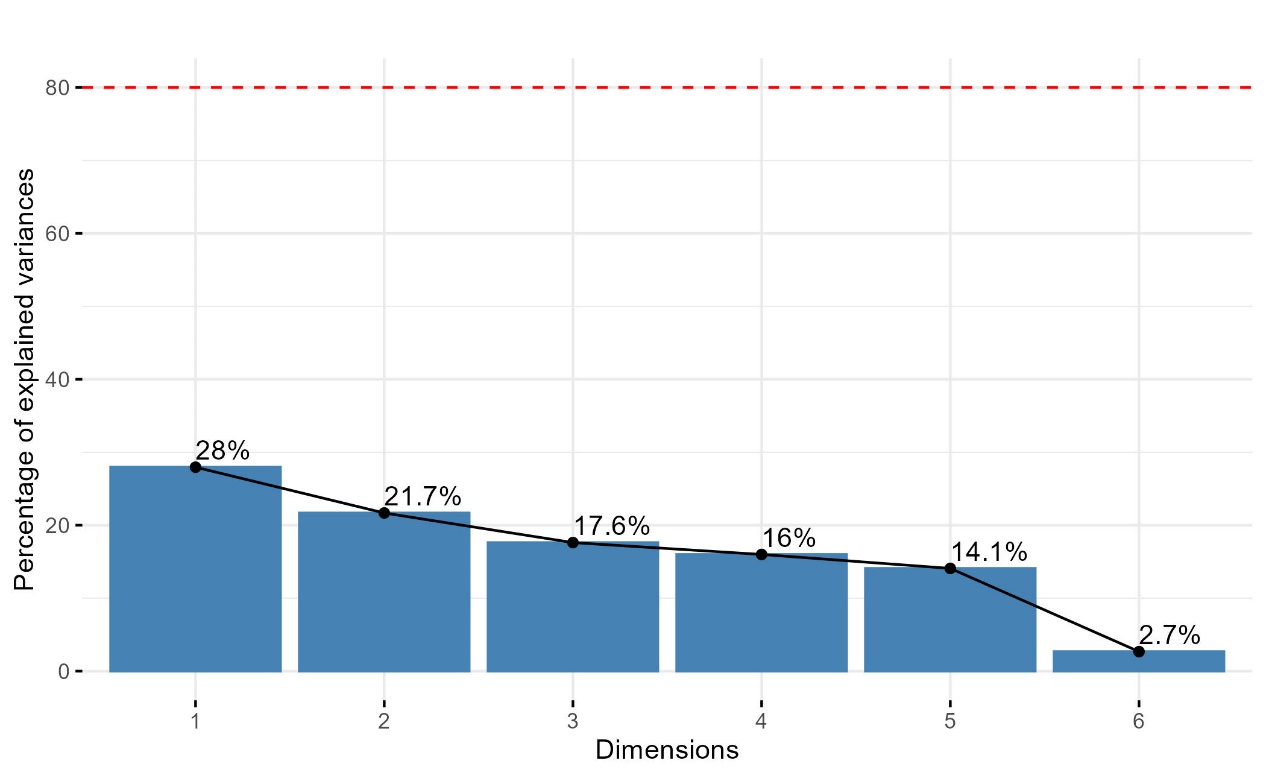


Figure S1 Scree Plot of Multiple Correspondence Analysis (MCA).


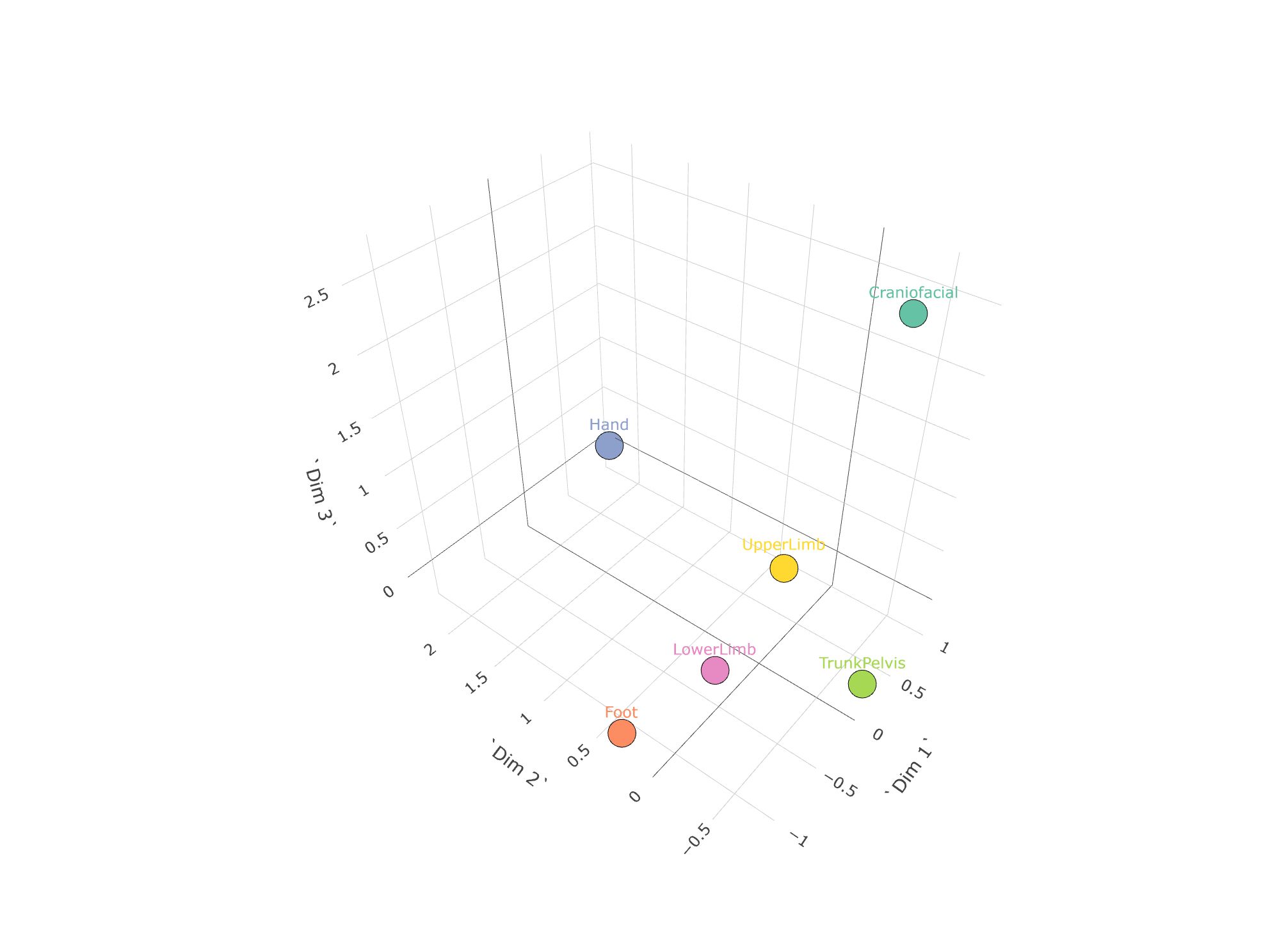


Figure S2 3D Variable Plot of Fracture Regions.
